# Meta-analysis of Multiple Myeloma and Benzene Exposure

**DOI:** 10.2188/jea.11.249

**Published:** 2007-11-30

**Authors:** Tomoko Sonoda, Yoshie Nagata, Mitsuru Mori, Tadao Ishida, Kohzoh Imai

**Affiliations:** 1 Department of Public Health, School of Medicine, Sapporo Medical University.; 2 First Department of Internal Medicine, School of Medicine, Sapporo Medical University.

**Keywords:** multiple myeloma, meta-analysis, benzene, engine exhaust

## Abstract

Epidemiologic studies have suggested that benzene exposure may be a risk factor of multiple myeloma (MM). We performed meta-analyses of case-control studies to assess the association between occupational exposure to benzene and the risk of MM. We divided the occupational sources of benzene exposure into 4 categories, benzene and/or organic solvents, petroleum, petroleum products, and engine exhaust, for conducting the meta-analysis. As a result, a significant positive association was indicated between exposure to engine exhaust and MM (summary odds ratio or summary OR=1.34, 95% confidence interval or 95%CI=1.14-1.57). However, no significant associations were obtained for benzene and/or organic solvents (summary OR=0.74, 95%CI=0.60-0.90), petroleum (summary OR=1.11, 95%CI=0.96-1.28) and petroleum products (summary OR=1.08, 95%CI=0.89-1.33) with risk of MM. These results suggested that benzene exposure itself was not likely to be a risk factor of MM. It is thought that several harmful chemical agents in engine exhaust, other than benzene, could be etiologically related to the risk of MM. Further case-control studies on MM are needed to obtain more information about detailed occupational exposure to toxic substances.

## INTRODUCTION

Multiple myeloma (MM) is a malignant proliferation of plasma cells in the bone marrow, and is characterized by lytic bone lesions, plasma cell accumulation in the bone marrow and the appearance of monoclonal proteins in the serum or urine. MM is more frequent in older people, and recently, its mortality rates have increased with the aging of the population^1^^)^. The survival experience is reported to be poorer for MM than for other cancers^2^^)^.

Epidemiologic efforts have suggested that several occupational sources are likely to be risk factors for MM. However, none of these sources have been etiologically confirmed as a cause of MM. Although it is well known that the bone marrow is damaged by chronic exposure to benzene^3^^)^, the positive association of benzene exposure with the risk of MM has not been consistently reported. Two hypotheses are considered as the reason for this inconsistency. One of them is the rarity of the disease as well as the small number of workers exposed to benzene. The other is the difficulty in getting information detailed levels and histories of benzene exposure in workers.

Because we plan to start an epidemiological study of MM in Hokkaido, Japan, we performed meta-analyses^4^^)^ of previously published studies on the association of the occupational benzene exposure with a risk of MM.

## MATERIALS AND METHODS

We searched the suitable articles using the Medline database from January 1966 through August 2000 under the following 4 criteria. 1) The article was either population-based or hospital-based case-control study. 2) The study reported the association between multiple myeloma and occupational or industrial exposure to benzene, petroleum, petroleum products, or engine exhaust. 3) The control was individually matched to each case at least on age and sex. 4) An Odds ratio (OR) and its confidence interval (CI) of exposure on MM was described. At last, 9 studies that filled all of the above conditions were selected.

Benzene is mainly included in petroleum and is discharged throughout the environment as engine exhaust^5^^, ^^6^^)^. So, occupational sources of benzene exposure were divided into the following 4 categories: ① benzene and/or organic solvents, ② petroleum, ③ petroleum products (rubber and/or plastic product), and ④ engine exhaust.

The homogeneity of the data in each exposure category was tested by the Q statistics based on the variance of ORs, and it was considered as statistically significant if p value was than 0.05. As a result, the homogeneity of the ORs in every exposure category was accepted.

There are the fixed effects model and the random effects model in the methods of data synthesis. In general, if the studies are homogenous, fixed and random effects models give similar results. We performed the data synthesis with Greenland’s method^16^^)^, which was one of the fixed effects model, because this method did not always require the numbers of exposed cases and controls.

The process of the Greenland’s method is as follows.

1) Standard error (SEj) = log (OR_j_ / lower 95%CI_j_) / 1.96
2) Weight of the study result (w_j_) = 1 / SE_j_^2^
3) Summary OR = exp[(∑w_j_×log OR_j_) / ∑w_j_]
4) 95%CI = exp [log (summary OR_j_)±(1.96×s)]
  (Where, s is the inverse of the square root of ∑w_j_.)

## RESULTS

**Figure 1.**
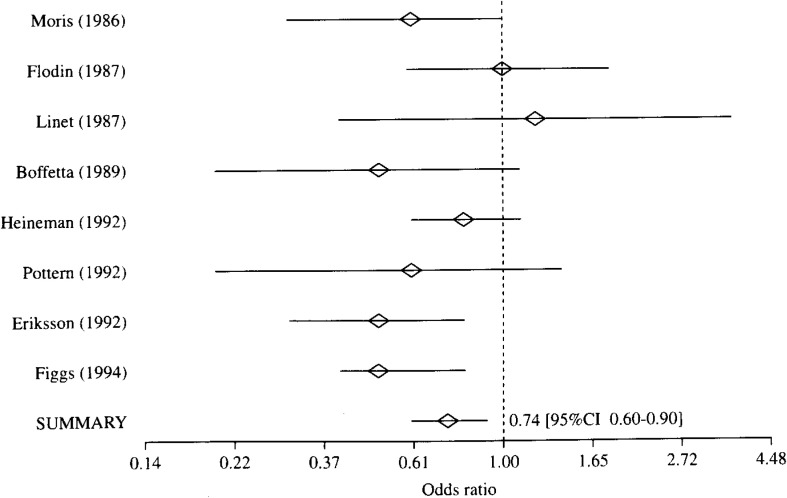
Result of meta-analysis of case-control studies on association between benzene and/or organic solvents and the risk of multiple myeloma.

Table 1 shows exposure substances, job titles, the numbers of cases and controls, and authors of each study, which are included to each exposure categories. Because the heterogeneity of the ORs in every exposure category was denied with the Q statistics, the meta-analyses were performed with Greenland’s method. The results are shown from Figure 1 to Figure 4. A logarithmic scale is used in order to exhibit these figures in a limited space.

**Figure 2.**
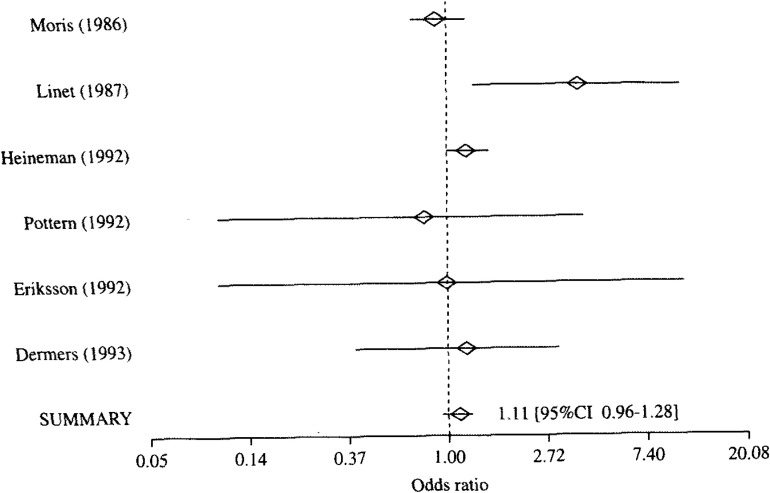
Result of meta-analysis of case-control studies on association between petroleum and risk of multiple myeloma .

**Figure 3.**
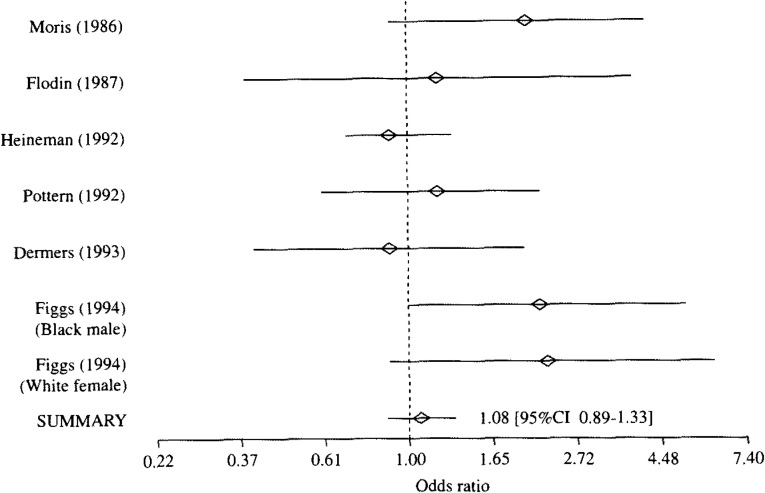
Result of meta-analysis of case-control studies on association between petroleum products and risk of multiple myeloma.

**Figure 4.**
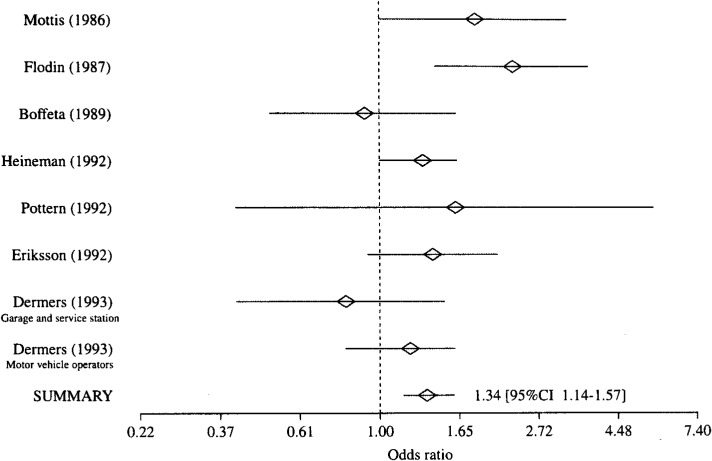
Result of meta-analysis of case-control studies on association between engine exhaust and risk of multiple myeloma.

**Table 1.** Exposure substances, job titles and the numbers of cases and controls according to exposure categories.

Exposure category	Substance or job title		No. of Cases	No. of Controls	Author
① Benzene and/or organic solvents	Aromatic hydrocarbons		698	1,683	Morris (1986)^7^^)^
	Solvents		131	431	Flodin (1987)^9^^)^
	Benzene		100	100	Linet (1987)^8^^)^
	Chemicals, Acids, Solvents		154	616	Boffetta (1989)^10^^)^
	Benzene		1,098	4,169	Heineman (1992)^11^^)^
	Organic solvents		1,010	4,040	Pottern (1992)^12^^)^
	Organic solvents		275	275	Eriksson (1992)^13^^)^
	Painter		12,148	60,740	Figgs (1994)^14^^)^

② Petroleum	Gasoline		698	1,683	Morris (1986)^7^^)^
	Petroleum		100	100	Linet (1987)^8^^)^
	Gasoline		1,098	4,169	Heineman (1992)^11^^)^
	Coal and oil product		1,010	4,040	Pottern (1992)^12^^)^
	Refinery worker		275	275	Eriksson (1992)^13^^)^
	Petroleum and coal refining and manufacturing		692	1,983	Dermers (1993)^15^^)^

③ Petroleum products	Plastic and rubber compounds		698	1,683	Morris (1986)^7^^)^
	Plastic-rubber chemicals		131	431	Flodin (1987)^9^^)^
	Chemical/rubber/plastic		1,098	4,169	Heineman (1992)^11^^)^
	Chemical/rubber/plastic		1,010	4,040	Pottern (1992)^12^^)^
	Rubber and plastic product manufacturing		692	1,683	Dermers (1993)^15^^)^
	Rubber and miscellaneous plastic products		12,148	60,740	Figgs (1994)^14^^)^
		(black male)			
	Rubber and miscellaneous plastic products		12,148	60,740	Figgs (1994)^14^^)^
		(white female)			

④ Engine exhaust	Automobile exhaust		698	1,683	Morris (1986)^7^^)^
	Engine exhaust		131	431	Flodin (1987)^9^^)^
	Gasoline exhaust		154	616	Boffetta (1989)^10^^)^
	Engine exhausts		1,098	4,169	Heineman (1992)^11^^)^
	Exhaust gases		1,010	4,040	Pottern (1992)^12^^)^
	Engine exhausts		275	275	Eriksson (1992)^13^^)^
	Garage and service station		692	1,683	Dermers (1993)^15^^)^
	Motor vehicle operators		692	1,683	Dermers (1993)^15^^)^

① Benzene and/or organic solvents (Figure 1): Of 8 studies evaluating these substances with regard to the risk of MM, 7 studies reported benzene or benzene-containing solvents as the exposure substances. Although one study^14^^)^ reported only job titles, other than the exposure substances, we selected painter as an occupation possibly involving exposure to organic solvents while working. As shown in Figure 1, the ORs were significantly decreased in 2 studies^13^^, ^^14^^)^. As a result of the meta-analysis, the summary OR of 8 studies was 0.74 (95%CI 0.60-0.90), and it was significantly decreased.

② Petroleum (Figure 2): Of 6 studies evaluating petroleum with regard to the risk of MM, 3 studies reported petroleum as the exposure substance. The other 3 studies reported workplaces exposed to petroleum such as those dealing with coal and oil products, refineries, and places of petroleum and coal manufacturing. As shown in Figure 2, the ORs were significantly increased in 2 studies^8^^, ^^11^^)^. However, the summary OR of 6 studies was 1.11 (95%CI 0.96-1.28), and it was not significant.

③ Petroleum products (rubber and/or plastic products) (Figure 3): All 7 studies evaluated workplaces producing rubber and/or plastic, other than exposure to petroleum products, with regard to the risk of MM. As shown in Figure 3, the OR was significantly increased in one study^14^^)^, especially, among black males. However, the summary OR of 7 studies was 1.08 (95%CI 0.89-1.33), and it was not significant.

④ Engine exhaust (Figure 4): Six studies evaluated engine exhaust as the exposure substances. One study^15^^)^ reported 2 workplaces, garage and service station, and motor-vehicle operating room, where workers are probably exposed to engine exhaust. As shown in Figure 4, the ORs were significantly increased in 3 studies^7^^, ^^9^^, ^^11^^)^. The summary OR of 7 studies was 1.34(95%CI 1.14-1.57), and it was statistically significantly elevated.

## DISCUSSION

Previous epidemiological research has suggested that occupational exposure to chemicals, radiation exposure, chronic allergic stimulation, and viral infection are the possible risk factors for MM. A number of studies have pointed out the relation of occupational exposure to pesticides, wood dust, organic solvents, petroleum, asbestos, metal, radiation, rubber, and plastic, to the risk of MM.

In addition, several studies reported the positive association of farming or agriculture with the risk of MM^6^^, ^^9^^, ^^10^^, ^^15^^, ^^18^^-^^25^^)^. Khunder^26^^)^ showed a significant positive association of farming with the risk of MM [relative risk (RR) =1.23, 95%CI 1.14-1.32] in his meta-analysis. He suggested that farmers are exposed to several agents, including solvents, viruses and other microbes, dust and agricultural chemicals, that might be associated with risk of MM. Except for farming, however, most occupational exposures have not been shown consistently to be risk factor for MM.

Benzene is included in petroleum and is discharged throughout the environment as engine exhaust. The result of our meta-analysis of exposure to engine exhaust indicated a significantly increased risk of MM (OR=1.34, 95%CI=1.14-1.57). Two hypotheses could be proposed for this significant result. The first is that the air concentrations of benzene are high in the workplaces of drivers or service-station workers, who are frequently exposed to engine exhaust. The air concentrations of benzene are estimated to be 0.2µg/m^3^ in the country areas and 349µg/m^3^ in industrial areas with much traffic^27^^)^. The median value of the concentrations of benzene in the car is estimated to be 14.0µg/m^3^ and the concentrations of benzene outside the car are estimated to be under 1.8µg/m^3 ^^27^^)^. The air concentration of benzene while filling gas tanks is estimated to be on the order of 1ppm (3000µg/m^3^)^5^^)^. The second hypothesis is that engine exhaust includes several other harmful chemical agents such as benzopyrene, ethylene, toluene, xylene, formaldehyde, suspended particulate matter (SPM), and so on. Some of these agents, other than benzene, may be associated with the risk of MM, although the carcinogenic effect of these agents on hematopoietic cells is not well established.

The most common sources of personal exposures to benzene are cigarette smoking and riding in automobiles. Active and passive smoking are the most important sources of exposure in the general environment. Smokers typically have a breath concentration of benzene around 14µg /m^3^, while in nonsmokers it is around 2µg /m^3 ^^5^^)^. However, the association between smoking and risk of MM has not been indicated, as the relative risks of smoking for MM were not significant in most of studies^8^^-^^10^^, ^^28^^-^^31^^)^.

The results of our meta-analyses did not show an increased association of benzene or benzene-containing organic solvents, petroleum and petroleum products (rubber and/or plastic products) with the risk of MM. Wong^32^^)^ reported that risk of MM was not associated with benzene exposure in a cohort of Pliofilm workers, and that there was no exposure-response relationship. Similar results have been reported in the studies of other countries^33^^, ^^34^^)^. Wong and Raabe^35^^)^ reported the pooled-analysis of 22 cohort mortality studies of more than 250,000 petroleum workers and they suggested that there was no causal relation between MM and petroleum workers as well as no exposure-response relationship. Other studies^3^^, ^^6^^, ^^36^^, ^^37^^)^ also did not consistently report the positive association of benzene exposure with the risk of MM.

The results of our meta-analyses suggested that benzene exposure itself is not likely to be a risk factor of MM. Instead, several harmful chemical agents in engine exhaust, rather than benzene, may be etiologically related to the risk of MM. However, further case-control studies on MM are needed to focus on information about detailed exposure to occupational toxic substances.

The assessment of exposure substances in the studies used in our meta-analysis was as follows. Some researchers^8^^, ^^10^^, ^^13^^)^ used the questionnaires contained the questions regarding exposure substances. Other researchers^11^^, ^^12^^)^ used the industry/occupational codes, which were distributed among Danish industrial hygienists who assessed exposure to 20 substance categories and 27 specific substances. The rest of the researchers^14^^, ^^15^^)^ did not describe exposure substances. However, we could select the jobs with probable exposure to benzene or related exposure substances, from their papers.

We obtained the unexpected result that benzene and/or organic solvents significantly decreased the risk of MM (ORs=0.74, CI=0.60-0.90). But, we can not interpret this result at this moment, as Eriksson et al^13^^)^ or Figgs et al^14^^)^ also could not. We started a case-control study on the association between occupational exposure and MM in Hokkaido, Japan, in July 2001 and would provide the another assessment for this inverse relationship of benzene and/or organic solvents to the risk of MM in the future.

## References

[1] Fujimoto I, Hanai A, Hiyama T, Tsukuma H, Takasugi Y. Cancer incidence and mortality in Osaka 1963-1989. Sinohara Publisher Inc. Tokyo. 1993.

[2] Ohshima A, Tsukuma H, Ajiki W. Survival of cancer patients in Osaka 1975-1989. Sinohara Publisher Inc. Tokyo. 1998.

[3] Wada O, Benzene problem in occupational health. Occup Health Rev, 2000; 13 : 49-81 (in Japanese).

[4] Blair A, Burg J, Foran J, . Guidelines for application of meta-analysis in environmental epidemiology. ISLI Risk Science Institute. Regul Toxicol Pharmacol, 1995; 22 : 189-197. 8577954 10.1006/rtph.1995.1084

[5] Wallace LA. Major source of benzene exposure. Environ Health Perspect, 1989; 82 : 165-169. 2477239 10.1289/ehp.8982165 PMC1568130

[6] Lynge E, Anttila A, Hemminki K. Organic solvents and cancer. Cancer Causes Control, 1997; 8 : 406-419. 9498902 10.1023/a:1018461406120

[7] Morris PD, Koepsell TD, Dalling JR, . Toxic substance exposure and multiple myeloma: a case-control study. J Natl Cancer Inst, 1986; 76 : 987-994. 3458965

[8] Linet MS, Harlow SD, McLaughlin JK. A case-control study of multiple myeloma in whites: chronic antigenic stimulation, occupation, and drug use. Cancer Res, 1987; 47 : 2978-2981. 3567914

[9] Flodin U, Fredriksson M, Persson B. Multilple myeloma and engine exhausts, fresh wood, and creosote: a case-control study. Am J Ind Med, 1987; 12 : 519-529. 2446496 10.1002/ajim.4700120506

[10] Boffetta P, Stellman SD, Garfinkel L. A case-control study of multiple myeloma nested in the American Cancer Society prospective study. Int J Cancer, 1989; 43 : 554-559. 2703267 10.1002/ijc.2910430404

[11] Heineman EF, Olsen JH, Pottern LM, . Occupational risk factors for multiple myeloma among Danish men. Cancer Causes Control, 1992; 3 : 555-568. 1420859 10.1007/BF00052753

[12] Pottern LM, Heineman EF, Olsen JH, Raffn E, Blair A. Multiple myeloma among Danish women: employment history and workplace exposures. Cancer Causes Control, 1992; 3 : 427-432. 1525323 10.1007/BF00051355

[13] Eriksson M, Karlsson M. Occupational and other environmental factors and multiple myeloma: a population based-control study. Br J Ind Med, 1992; 49 : 95-103. 1536825 10.1136/oem.49.2.95 PMC1012073

[14] Figgs LW, Dosemeci M, Blair A. Risk of multiple myeloma by occupation and industry among men and women: a 24-state death certificate study. J Occup Med, 1994; 36: 1210-1221. 7861265 10.1097/00043764-199411000-00007

[15] Demers PA, Vaughan TL, Koepsell TD, . A case-control study of multiple myeloma and occupation. Am J Ind Med, 1993; 23 : 629-639. 8338527 10.1002/ajim.4700230410

[16] Greenland S. Quantitative methods in the review of epidemiologic literature. Epidemiol Rev, 1987; 9 : 1-30. 3678409 10.1093/oxfordjournals.epirev.a036298

[17] Gallagher RP, Spinelli JJ, Elwood JM, Skippen DH. Allergies and agricultural exposure as risk factors for multiple myeloma. Br J Cancer, 1983; 48 : 853-857. 6652026 10.1038/bjc.1983.277 PMC2011571

[18] Franceschi S, Barbone F, Bidoli E, . Cancer risk in farmers: results from a multi-site case-control study in north-eastern Italy. Int J Cancer, 1993; 53 : 740-745. 8449597 10.1002/ijc.2910530506

[19] Blair A, Dosemeci M, Heineman EF. Cancer and other causes of death among male and female farmers from twenty-three states. Am J Ind Med, 1993; 23 : 729-742. 8506851 10.1002/ajim.4700230507

[20] Burmeister LF, Everett GD, Van Lier SF, Isacson P. Selected cancer mortality and farm practices in Iowa. Am J Epidemiol, 1983; 118 : 72-77. 6869365 10.1093/oxfordjournals.aje.a113618

[21] Cantor KP, Blair A. Fanning and mortality from multiple myeloma: a case-control study with the use of death certificates. J Natl Cancer Inst, 1984; 72 :251-255. 6582313

[22] Inskip H, Coggon D, Winter P, Pannett B. Mortality of farmers and farmers’ wives in England and Wales 1979-80, 1982-90. Occup Environ Med, 1996; 53 :730-735. 9038795 10.1136/oem.53.11.730 PMC1128589

[23] Pearce NE, Smith AH, Howard JK, . Case-control study of multiple myeloma and farming. Br J Cancer, 1986; 54 :493-500. 3756085 10.1038/bjc.1986.202 PMC2001629

[24] Steineck G, Wilkund K. Multiple myeloma in Swedish agricultural workers. Int J Epidemiol, 1986; 15 :321-325. 3771067 10.1093/ije/15.3.321

[25] Viel J, Richardson ST. Lymphoma, multiple myeloma and leukemia among French farmers in relation to pesticide exposure. Soc Sci Med, 1993; 37 :771-777. 8211293 10.1016/0277-9536(93)90371-a

[26] Khuder SA, Mutgi AB. Meta-analysis of multiple myeloma and farming. Am J Ind Med, 1997; 32 : 510-516. 9327075 10.1002/(sici)1097-0274(199711)32:5<510::aid-ajim11>3.0.co;2-5

[27] Environmental Health Criteria No.150 Benzene. WHO, 1993

[28] Brown LM, Everett GD, Burmeister LF, Schuman LM, Blair A. Smoking and risk of non-Hodgkin’s lymphoma and multiple myeloma. Cancer Causes Control, 1992; 3 : 49-55. 1536913 10.1007/BF00051912

[29] Miligi L, Seniori Costantini A, Crosignani P, . Occupational, environmental, and life-style factors associated with the risk of hematolymphopoietic malignancies in women. Am J Ind Med 1999; 36: 60-69. 10361588 10.1002/(sici)1097-0274(199907)36:1<60::aid-ajim9>3.0.co;2-z

[30] Herrinton LJ, Koepsell TD, Weiss NS. Smoking and multiple myeloma. Cancer Causes Control, 1992; 3 : 391-392. 1617129 10.1007/BF00146895

[31] Williams RR, Horm JW. Association of cancer sites with tobacco and alcohol consumption and socioeconomic status of patients: interview study from the Third National Cancer Survey. J Natl Cancer Inst, 1977; 58 : 525-547. 557114 10.1093/jnci/58.3.525

[32] Wong O. Risk of acute myeloid leukemia and multiple myeloma in workers exposed to benzene. Occup Environ Med, 1995; 52 : 380-384. 7627314 10.1136/oem.52.6.380 PMC1128241

[33] Paci E, Buiatti E, Seniori Costantini AS, . Aplastic anemia, leukemia and other cancer mortality in a cohort of shoe workers exposed to benzene. Scand J Work Environ Health, 1989; 15 :313-318. 2799316 10.5271/sjweh.1845

[34] Yin SN, Hayes RB, Linet MS, . A cohort study of cancer among benzene-exposed workers in China: overall results. Am J Ind Med, 1996; 29 : 227-235. 8833775 10.1002/(SICI)1097-0274(199603)29:3<227::AID-AJIM2>3.0.CO;2-N

[35] Wong O, Raabe GK. Multiple myeloma and benzene exposure in a multinational cohort of more than 250,000 petroleum workers. Regul Toxicol Pharmacol, 1997; 26 : 188-199. 9356282 10.1006/rtph.1997.1162

[36] Rinsky RA, Smith AB, Hornung R, . Benzene and leukemia. An epidemiologic risk assessment. N Engl J Med, 1987; 316: 1044-1050. 3561457 10.1056/NEJM198704233161702

[37] Bergsagel DE, Wong O, Bergsagel PL, . Benzene and multiple myeloma: appraisal of the scientific evidence. Blood, 1999; 94 : 1174-1182. 10438704

